# Efficacy and safety of transepithelial collagen cross linking for progressive keratoconus

**DOI:** 10.12669/pjms.325.10922

**Published:** 2016

**Authors:** Sameer Shahid Ameen, Mohammad Asim Mehboob, Kashif Ali

**Affiliations:** 1 Prof. Sameer Shahid Ameen, MCPS, FCPS. Department of Ophthalmology, PNS Shifa Naval Hospital, Karachi, Pakistan; 2 Dr. Mohammad Asim Mehboob, MBBS. Department of Ophthalmology, PNS Shifa Naval Hospital, Karachi, Pakistan; 3 Dr. Kashif Ali, FCPS. Department of Ophthalmology, PNS Shifa Naval Hospital, Karachi, Pakistan

**Keywords:** Keratoconus, Collagen Cross linking, CXL

## Abstract

**Objective:**

To evaluate the efficacy and safety of transepithelial (TE) collagen cross-linking (CXL) in patients with progressive keratoconus (KC).

**Methods:**

This Quasi Experimental Study was conducted at PNS Shifa Naval Hospital, Karachi from June 2015 to June 2016. Sixty eyes of 32 patients who underwent TE CXL for progressive KC from June 2015 to June 2016 were analysed to ascertain efficacy and safety of TE CXL procedure. Statistical analysis of the data was done using SPSS version 17.0.

**Results:**

Twenty eight (87.5%) patients underwent TE CXL bilaterally, while 4 (12.5%) underwent unilateral CXL. Mean change in astigmatism, Maximum simulated Keratometry value (Kmax), Spherical equivalent (SE) and Central Corneal Thickness (CCT) were -0.67±0.35D, 1.28±0.64D, -0.58±0.17D and 0.40±7.58µm respectively, from baseline. Mean gain in lines on Snellen’s visual acuity chart was 1.13±0.83 lines. Changes in astigmatism, Kmax and SE were statistically significant (p<0.001), while change in CCT was not statistically significant. The procedure had excellent safety profile, with no major complication till 6 months follow up period.

**Conclusion:**

TE CXL is a safe and effective procedure with statistically significant reduction in corneal astigmatism, Kmax and SE with reasonable gain in Snellen’s visual acuity.

## INTRODUCTION

Cornea is the most significant contributor to clear image formation by human optical system. Diseases affecting cornea cause a significant reduction in vision and vision related quality of life. Keratoconus (KC) is a corneal degenerative condition with morphological changes in corneal stroma leading to thinning, and change to a more conical shape than the normal gradual curve.^1^ The condition affects early age groups, with majority of patients presenting in 2nd or 3rd decade. The prevalence of KC in general population has been variable, ranging from 50 to 200 per 100 000.^2^ Patients with KC suffer from visual deterioration due to myopia, high and irregular astigmatism, corneal opacification and scarring. The condition is managed conservatively using glasses and rigid contact lenses. Recent management options include intra corneal stromal ring segments, with a significant number of patients eventually requiring penetrating keratoplasty due to scarring and extreme corneal ectasia.^3^ Corneal collagen cross-linking (CXL) is a novel method for halting progression of KC using riboflavin and Ultraviolet A (UVA) to strengthen the collagen of corneal stroma. The procedure was introduced in animal models in 1980’s, while Wollensak et al first reported the efficacy, safety and biocompatibility of the procedure in human corneas.^4^ Since then, the procedure has seen scientific evolution with newer and efficient methods, protocols and techniques for better safety and efficacy. The standard method initially described by Wollensask et al included initial epithelial removal, application of riboflavin solution for 30 minutes with subsequent UVA irradiation for 30 minutes.^5^ The procedure had side effects related to epithelial removal and healing. This lead to emergence of trans epithelial (TE) CXL with modification in riboflavin formulation for better penetration through intact corneal epithelium. The literature shows significant fewer side effects, specially related to epithelium, but has also undermined the efficacy of the process.^6^,^7^ Randomized control trials have highlighted less side effects, but also lesser efficacy of the TE CXL procedure, keeping the debate of its utility alive.^8^ The aim of this study was to evaluate the efficacy and safety of TE CXL in Pakistani population of patients with progressive KC.

## METHODS

After approval by the hospital ethical review committee, informed written consent was taken from the patients prior to inclusion in the study. A sample size of ten eyes was required to detect a difference of 0.75 D between the Maximum simulated Keratometry value (Kmax), 12 months after treatment and at baseline, at a significance level of 0.05 and a power of 80%, assuming a standard deviation of 0.75 D.^9^ Since our follow up time was 6 months, we increased the sample size, to also compensate for patients losing follow up. Patients aged between 18-30 years, with best corrected visual acuity (BCVA) of less than 6/6 on Snellen’s visual acuity chart, central or inferior steepning, documented clinical and instrumental (topographic, pachymetric) worsening of astigmatism, KC stage 1-3 according to Amsler Krumeich Staging of KC, central corneal thickness of (CCT) more than 400 microns, and willing to follow up for minimum of 6 months were included. Patients with corneal scarring, previous history of trauma, rigid contact lens use or herpetic keratitis, pregnancy, lactation, nystagmus and any diagnosed autoimmune diseases were excluded.

Clinical pre-operative testing included slit-lamp exam and measurement of distant visual acuity, BCVA, spherical error, corneal astigmatism, spherical equivalent (SE), Kmax and CCT using Specular Microscope (Topcon SP 3000P, Topcon Corporation, Tokyo, Japan).

### Procedure

All patients received proparacaine with benzalkonium chloride (BAK) 0.01% every 5 minutes for 20 minutes to facilitate riboflavin absorption. Riboflavin (Vitamin B2) 0.25 %, 1.2 % hydroxypropyl methylcellulose (HPMC), BAK 0.01 % (Peschke TE, Peschke Meditrade GmbH, Huenenberg, Switzerland) available in pre-loaded glass syringe containing 2.0 ml liquid was instilled, one drop every two minutes for 20 minutes with proparacaine 0.01%, 1 drop every 10 minutes. Stromal saturation was confirmed using slit lamp. All eyes then received UVA 365 nm radiation for 10 minutes at irradiance of 9mW/cm2 using Peschke CXL system (CCL-Vario Crosslinking; Peschke Meditrade GmbH, Zurich, Switzerland). Post procedure, all patients received topical Moxifloxacin 0.5%, 6 hourly for 7 days and Prednisolone acetate 1%, 8 hourly for two weeks. The patients were reviewed on first post-operative day, after 7 days, and monthly for 6 months. All procedures were performed by single surgeon to exclude bias. The principal outcomes included change in astigmatism, Kmax, SE, CCT and gain in number of lines on Snellen’s visual acuity chart. The safety was evaluated for symptoms of pain, photophobia, and signs like epithelial erosions, corneal ulcer, uveitis and raised intraocular pressure (IOP). A questionnaire regarding subjective pain scoring (Table-I) was filled, and the pain score was calculated for each patient. The pre devised proforma was completed by single researcher endorsing subject’s demography, ocular examination findings and outcome measures.

**Table-I:** Subjective Pain Score Proforma.

*Score*	*Intensity*	*Description*
0	No pain	No pain or discomfort
1	Very Mild	Mild discomfort
2	Mild	Mild pain, Did not require consultation
3	Moderate	Moderate pain, required consultation, Tear substitutes
4	Severe	Severe pain, Required oral analgesia
5	Very Severe	Severe pain, required oral analgesia and topical anaesthesia

### Statistical Analysis

Statistical package for social sciences (SPSS 17.0) for windows was used for statistical analysis. Descriptive statistics i.e. mean ± standard deviation for quantitative values (age, astigmatism, Kmax, SE, CCT, gain in number of lines) and frequencies along with percentages for qualitative variables (gender, laterality of eyes, complications) were used to describe the data. Shapiro Wilk’s test was used to check normality of data. Post normality testing, Paired ‘t’ test was used to compare pre-operative astigmatism, Kmax, SE, and CCT from post-operative values. P value < 0.05 was considered statistically significant.

## RESULTS

Sixty eyes of 32 patients fulfilling the inclusion criteria were analysed. Mean age of study population was 23.78 ± 3.03 years (Range 18-29 years). Out of 32 patients, 17 (53.1%) were male, while 15 (46.9%) were females. 28 (87.5%) patients underwent TE CXL bilaterally, while 4 (12.5%) underwent unilateral CXL.

### Efficacy

Mean pre-operative and post-operative astigmatism, Kmax, SE, and CCT along with mean change are given in Table-II. Change in astigmatism, Kmax and SE from baseline was statistically significant (p<0.001), while change in CCT was not statistically significant (p=0.685). Mean gain in lines on Snellen’s visual acuity chart was 1.13±0.83 lines. 17(28.3%) patients did not gain any line on Snellen’s chart. 18 (30%) patients gained one line, while 25 (41.7%) gained two or more lines.

**Table-II:** Results of TE CXL (n=60).

*Parameter*	*Pre-op Value*	*Post-Op Value*	*Mean Change*	*p Value*
Kmax (D) Mean ± SD	53.14 ± 1.81	51.86± 1.58	1.28 ± 0.64	< 0.001
Astigmatism (D) Mean ± SD	-3.72 ± 0.35	-3.04 ± 0.45	0.67 ± 0.35	< 0.001
SE (D) Mean ± SD	-5.86 ± 0.17	-5.27 ± 0.22	0.58 ± 0.17	< 0.001
CCT (µm) Mean ± SD	447.88 ± 27.93	448.28± 27.81	0.40 ± 7.58	0.685

### Safety

**Fig.1:**
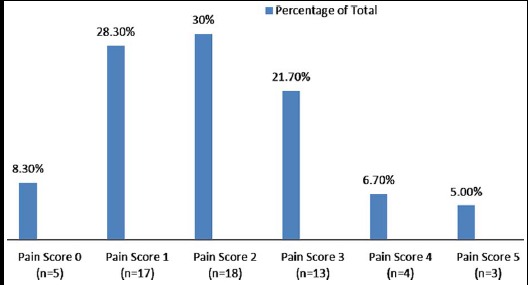
Frequency of Pain Score (n=60).

**Fig.2:**
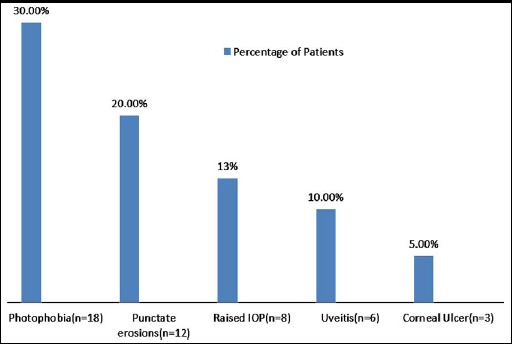
Complications TE CXL (n=60).

The mean pain score for all patients was 2.05±1.25. Frequency of pain score for all patients is illustrated in Fig.1. Most frequent complication noted was photophobia in 18(30%) patients. Other less frequent and reversible complications are given in Fig.2. The procedure has excellent safety profile, with no major complication till 6 months follow up period.

## DISCUSSION

Management of KC is a daunting task for ophthalmologists. Most of the treatment strategies for KC are focused at treating astigmatism and improving visual acuity. However, CXL provides a unique method to impede the progression of KC by using UVA radiation. We used irradiation time of 10 minutes, at irradiance of 9mW/cm2 using Peschke CXL system. Few studies have shown better results with standard 30 minutes irradiation protocol, as recommended by Wollensak et al initially.^10^,^11^ However, accelerated CXL has also shown promise, which uses higher irradiance in lesser time to obtain desired results.^12^ Kir MB et al in their study on 48 eyes showed stabilization of BCVA and corneal topographical indices with irradiance of 45mW/cm2 for two minutes after two years.^13^ Cummings AB et al also showed more flattening of cornea using accelerated CXL as compared to standard CXL.^14^ Our study also shows comparable results using accelerated TE CXL protocol.

The conventional CXL needed epithelium removal for better penetration of riboflavin in corneal stroma. The penetration of intact corneal epithelium is difficult as riboflavin is chemically a hydrophilic molecule. Researches have proved that the cornea retains its physiological properties when CXL is performed with intact epithelium.^15^ Other approaches like iontophoresis enhance delivery of hydrophilic riboflavin to deeper corneal collagen using small, low voltage electric current.^16^ Other methods to improve corneal penetration are chemical disruption using BAK or tetracaine, use of hypotonic riboflavin without dextran, and slight increase in hypotonic riboflavin concentration from 0.1% to 0.5% to utilize increased osmotic gradient.^17^ we used hypotonic riboflavin 0.25%, with HPMC and BAK to utilize both osmotic gradient and chemical disruption technique for better corneal penetration. The saturation of cornea with riboflavin was checked using slit lamp and slight flare in the anterior chamber confirmed before application of UVA light.

Our study reported significant reduction in Kmax, SE and astigmatism with significant improvement in BCVA. 71.7% of our patients gained one line or more on Snellen’s visual acuity chart, while 28.3% didn’t gain any line. Importantly, none of the patient lost any line during 6 monthly follow up time. TE CXL is novel method that can be used effectively in patients with thin corneas, since epithelium is not peeled. Sine change in CCT was not significant, TE CXL also provides sufficient stability over 6 months follow up period. However, the efficacy of TE CXL has remained questionable in literature. Studies with one year follow up have confirmed stabilization of Kmax and BCVA in patients with KC.^7^,^18^ Other studies have shown decrease in Kmax and improvement in BCVA for a period after 18 months of TE CXL.^6^,^19^ Few studies have also reported continued worsening of Kmax and stabilization or decrease in BCVA, making the use of TE CXL debatable.^20^ Chen S et al in their study on 21 eyes who underwent TE CXL reported that a higher pre-operative Kmax correlated with greater corneal flattening after CXL, making TE CXL an impressive treatment option.^21^ Studies comparing TE CXL with standard CXL using randomized control trials have shown improvement in both Kmax and BCVA in both protocols. However, the change and stabilization was observed more and prolonged in those treated with standard CXL.^22^ Since the results of both TE and standard CXL are comparable, TE CXL offers a trade-off between better safety profile and clinically acceptable efficacy.

Standard CXL is associated with serious complications like bacterial keratitis, acute hydrops, persistent epithelial defect, neurotrophic keratopathy, and corneal melting.^23^ Post-operative complications are an important reason of shifting focus from standard epithelium Off CXL to TE CXL. Most significant complication after standard CXL was pain, which required placement of bandage contact lens and analgesia.^24^ Our study showed that post-operative pain was minimal in TE CXL patients with only 11.7% (Pain score 4 or more) requiring oral or topical analgesia. This is far less than the patients requiring analgesia in standard CXL.

The commonest complication noted was photophobia, which was observed in 30% (n=18) of the patients. The condition was transient in nature, with almost all patients improving to normal condition in two weeks post-operative follow up. Punctate erosions, uveitis and raised IOP were observed in 20%, 10% and 13.3% of the patients respectively. These complications were also transient in nature, with complete recovery in two weeks follow up period. The most severe complications was corneal ulcer formation in 5% patients, which required prolonged management. Fortunately, all patients recovered fully with no complications and restoration of pre-operative BCVA. All these complications were transient, making TE CXL a safe procedure.

### Limitations of the study

Shorter follow up time and no control group for comparison of safety and efficacy. Longer follow up is required to ascertain long term efficacy and safety of TE CXL and comparison with control group undergoing standard CXL will provide reliable and comparable results.

## CONCLUSION

Our study has highlighted that TE CXL is a safe and effective procedure with statistically significant reduction in corneal astigmatism, SE and Kmax with reasonable gain in Snellen’s visual acuity. There is need to carry out randomized control trials with longer follow up time to compare TE CXL with conventional epithelium Off CXL for better comparison of efficacy and safety of both procedures.
